# Cost-Effectiveness of Portable Electrocardiogram for Screening Cardiovascular Diseases at a Primary Health Center in Ahmedabad District, India

**DOI:** 10.3389/fpubh.2021.753443

**Published:** 2021-12-03

**Authors:** Komal Shah, Apurvakumar Pandya, Priya Kotwani, Somen Saha, Chintan Desai, Kirti Tyagi, Deepak Saxena, Tapasvi Puwar, Shilpa Gaidhane

**Affiliations:** ^1^Regional Resource Centre for Health Technology Assessment, Indian Institute of Public Health, Gandhinagar, India; ^2^District Panchayat, Department of Health, Ahmedabad, India; ^3^Department of Health Research, Ministry of Health and Family Welfare, New Delhi, India; ^4^Department of Medicine, J. N. Medical College, Datta Meghe Institute of Medical Sciences, Wardha, India

**Keywords:** health technology assessment (HTA), cost-effectiveness (CE), portable electrocardiogram devices, primary health center-PHC, India, Asia—Pacific

## Abstract

**Background:** District Health Authority in Ahmedabad, Gujarat has introduced Project Lifeline, 12-lead portable ECG devices across all primary health centers (PHC) in the district to screen cardiac abnormalities among high-risk and symptomatic adults for providing primary management and proper timely referral. The prime purpose of the study was to assess the cost-effectiveness of portable ECG for the screening of cardiovascular diseases (CVD) among high-risk and symptomatic adults at the PHC in Ahmedabad, Gujarat.

**Methods:** Cost-effective analysis was conducted using a societal perspective. An incremental costing approach was adapted, and cost-effectiveness analysis was done using a decision-analytic model. We surveyed 73 patients who screened positive for cardiac abnormality, documented the type of ECG abnormalities, and diagnosed CVD. The program cost was obtained from the implementers. Transition probabilities were derived from primary data supported by expert opinion for the intervention arm, while a systematic search of the literature was undertaken to derive transition probabilities for the control arm.

**Results:** The ECG screening at PHC saves 2.90 life years at an incremental cost of 89.97 USD (6657.47 INR), yielding a cost-effectiveness ratio of 31.07 USD (2,299.06 INR) per life-year saved, which is below the willingness to pay threshold. The budget impact analysis was also performed. Results are sensitive to the relative risk reduction associated with the non-participation and the cost of initial screening.

**Conclusion:** Cost-effectiveness analysis clearly shows that the facility to screen cardiac abnormality at the PHC level is highly recommended for high-risk adults and symptomatic cases.

## Introduction

Cardiovascular diseases (CVD) are emerging as the number one cause of death across the globe. Globally 70% of all deaths are due to non-communicable diseases (NCDs) (1). In India, 26% risk of death can be attributed to CVDs. About 23% of those with heart attacks do not survive due to delay in treatment leading to the death of around 1.7 million Indians (2).

Considering the silent progression of the disease and the requirement of specific expertise for diagnosis and treatment, early diagnosis and treatment facilities are extremely limited at primary health centers (PHCs). Advancement in diagnostic methods has provided handheld portable electrocardiography (ECG) technology that can effectively screen some cardiac abnormalities in the absence of conventional ECG machines, especially at the PHC level. Prompt screening, early identification of true cases and prompt management, especially with thrombolytic and aspirins with timely referral in “GOLDEN HOUR” (the first 60 min of a heart attack) is, of utmost importance.

Usage of portable ECG facilities in various forms such as single lead and 12-lead handheld instruments has been studied by many for effective management and early identification of cardiac abnormalities in various health care settings (3–5). It was found to be a cost- and clinically effective strategy of screening in patients of atrial fibrillation and elderly population (>70 years) as it significantly reduces the risk of stroke and any other cardiac event due to early diagnosis and management (3). Economic evaluation studies reported that opportunistic screening for atrial fibrillation in primary care has the potential to be cost-effective (3, 6). However, the competency of primary care practitioners and nurses for interpreting the ECG readings needs to be considered for the successful implementation of such a screening program. Begg et al. in their work suggested that primary care practitioners were less experienced and less confident with ECG interpretation than cardiologists and require support in interpreting ECG readings (7). In scenarios with limited capabilities, solutions such as telecardiology (bringing expert ECG interpretation to primary care) can save time, money, and lives. Both primary care physicians (PHC-Medical Officer—PHC-MO) and patients benefit from the ease of access, speed of diagnosis, management efficiency, and the freeing up of resources (8). As PHC-MOs remain the main point of contact within the primary health care system for most of the population, they play an instrumental role in the early detection and management of CVDs.

In Gujarat, the Government has established ECG facilities, but it is limited to medical colleges (MC), district hospitals (DH), and subdistrict hospitals (SDH), and Community Health Centers (CHC). PHCs are not yet equipped with these facilities. To screen all the high-risk and symptomatic adults, the District Health Authority in Ahmedabad, Gujarat, has introduced a 12-lead portable ECG machine across 40 PHCs in the district for the first time in the state. Linkage was established with a cardiologist to read ECG through a web-based interface (WhatsApp) to identify and confirm CVDs and provide primary management (with thrombolytic and antiplatelet such as Aspirin) coupled with a timely referral. For timely ECG reading and guidance from cardiologists, the incentive of 0.41 USD (30 INR) per reading (per case) was provisioned in the program. Under this initiative, PHC-MOs were trained using the cascade model, wherein these PHC-MOs then trained the PHC staff within 3 days of receiving the ECG device at the PHCs. The District Health Authority, Ahmedabad, requested the Regional Resource Center for Health Technology Assessment at the Indian Institute of Public Health Gandhinagar to conduct a cost-effectiveness study of the Project Lifeline.

The objective of the present work was to assess the cost-effectiveness of introducing a portable ECG facility at PHCs for the screening of CVD among high-risk and symptomatic adults, and to estimate budgetary implications for the scale-up of the ECG facility. The Technical Advisory Committee at the Department of Health Research, Government of India, approved the study.

## Materials and Methods

**Figure 1:**
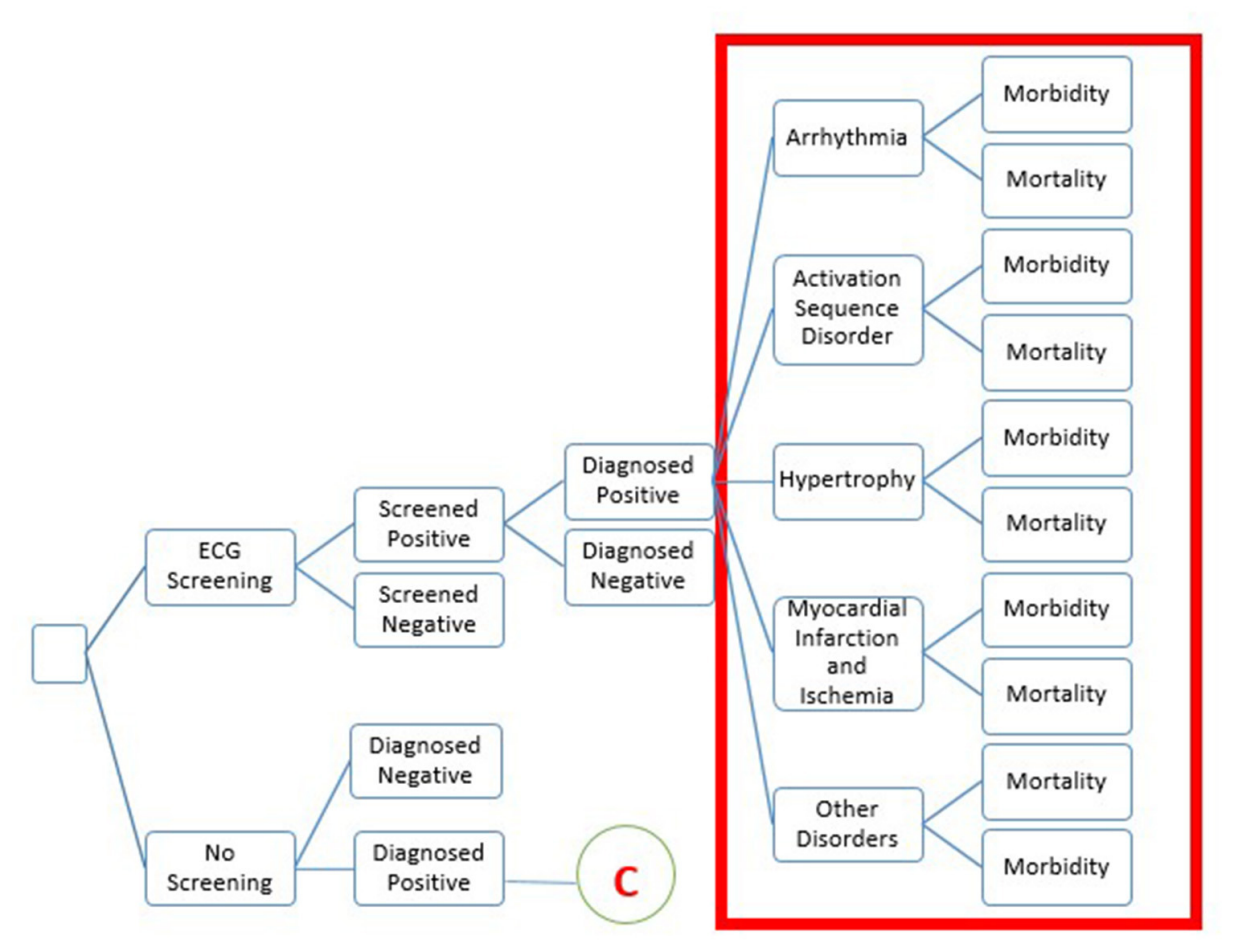
The structure of decision-tree model. The decision tree describes the pathway of a patient presenting to primary care with signs or symptoms who will be screened using the ECG device. Diagnosed positive cases will be further classified into five sets of diseases. During this period, some patients will have a diagnosis and start treatment or die. The second arm, no screening will be considered to follow similar pathways for diagnosed positive.

Cost-effectiveness analysis was done using decision-analytic modeling (Figure 1) with a societal perspective on health care costs and benefits.

A decision tree was parameterized on the Microsoft-Excel spreadsheet to estimate the incremental cost-effectiveness ratio. The target population for the study was high-risk and symptomatic adults, which included adults having diabetes, hypertension, cardio-metabolic syndrome, family history of cardiac disease, or signs and symptoms suggestive of CVD. Intervention scenario, viz. screening of population with portable ECG machine for early detection of cardiac abnormalities at the PHC, was compared with no routine care scenario. Early diagnosis, prompt treatment of CVD, and life-years saved were the outcome measures. The number of patients screened using a portable ECG device and the number of patients identified with abnormality were derived from the secondary data maintained at District Panchayat, Ahmedabad.

The ECG abnormalities identified during the screening were categorized into five major disorders based on the primary data and expert opinion. The five cardiovascular disorders reported in the high-risk adults mentioned in Table 1 were considered for building the decision tree model. We have accessed the data of 12,105 individuals who visited PHC screened for CVD using portable ECG devices during the period from 2018 to 2019. Of this, 208 individuals screened with abnormalities were selected from the OPD database maintained at the District Panchayat, Ahmedabad. Of the patients screened positive, 127 were pregnant women, 73 were high-risk symptomatic adults, and 10 were children. Since the objective of this work was to assess the cost-effectiveness of screening high-risk and symptomatic adults in a primary healthcare setting, the analysis reported here is restricted to this particular population. Further follow-up of 73 high-risk symptomatic adults led to 54 individuals who were diagnosed positive for CVD. Table 1 categorized the ECG abnormalities based on expert opinion.

**Table 1:** Categorization of ECG abnormalities based on expert opinion.

**Cardiovascular disorders**	**ECG abnormalities**
Arrhythmia	•Supraventricular arrhythmia •Ventricular arrhythmia
Action sequence conduction defect	•Atrioventricular conduction defect (block) •Bundle branch block
Increase in wall thickness or size of atria or ventricles	•Atrial hypertrophy •Ventricular hypertrophy
Myocardial ischemia	•Myocardial ischemia or infarction
Others	•Valvular issues

### Assumptions Used for Modeling

There are data gaps in screening at the primary care facilities. Therefore, we had no relevant comparator. We compared screening with routine treatment scenarios where CVD abnormalities are not currently performed at the primary care level.

The purpose of introducing ECG was to enable the early identification of CVD abnormalities, thereby preventing severe morbidity and mortality. Due to the lack of relevant data on outcomes of screened negative and diagnosed negative in both arms, we could not demonstrate it in modeling, which is a limitation of the study. We tried finding evidence of clinical validity of the diagnostic tests for specific abnormalities identified through a systematic review of the literature, but could not find it. Hence, all screened positive cases were subjected to the gold standard diagnostic test to predict the disease to establish the clinical validity of the screening test.

The number of people diagnosed with the screening ECG is lower than in the non-screening arm as well as differences in the age group in both arms are evident. This is because the data of the intervention arm reflects the data collected from the primary study where patients were followed-up for the diagnosis, whereas in the comparator arm data was taken from CREATE registry of India, which is available in the public domain. This data reflects the population who consulted for diagnosis on the occurrence of severe symptoms and when the disease is expressed.

### Derivation of the Cost Data

As the study adopted using a societal perspective, both the program cost, i.e., the cost borne by the health system for implementing the ECG program and the direct and indirect medical cost incurred by the patients were considered. The program cost was obtained from the implementers under two cost heads, capital cost and annual implementation cost. Capital costs included start-up costs such as ECG equipment and orientation training cost since the launch of the program. The capital cost, including start-up cost, was annualized, assuming the life year of the ECG device to be 10 years. The recurrent costs consisted of the annual maintenance cost, incentives provided to physicians for interpretation of ECG reading, shared human resource cost, and other contingency costs such as meeting, training of PHC staff by MO, and cost of ECG screening materials (prints, cartridge, etc.). To estimate the programmatic cost, financial records of District Panchayat, Ahmedabad were used and a time-motion study was undertaken to estimate the shared human resource cost.

All costs were reported in Indian Rupees and USD at 74 INR per dollar. In addition to the programmatic cost, the out-of-pocket expenditure (OOPE) of the patient was estimated using published literature (9), which comprised of cost of medications, transportation, wage loss of the patient, and the caretaker. Both the recurrent and capital costs were collected and summed up to arrive at the total cost. All costs were presented in INR. Costs were converted to constant values and reported as annualized costs in the 2018–2019 price.

For deriving the cost of treatment, a group of physicians were consulted for their opinion on the line of treatment. The cost of interventions (as suggested by the experts) were taken from Pradhan Mantri Jan Arogya Yojana (PMJAY) Package (10). Since the cost for undergoing a diagnostic test was already included in the PMJAY, we have not added additional diagnostic costs to avoid overcalculation of the treatment cost.

### Derivation of Data on Transition Probabilities

Transition probabilities were derived from primary data supported by expert opinion for the intervention arm, and a systematic literature search was undertaken to derive transition probabilities for the control arm. Three experts included two prominent cardiologists from Gujarat and one community medicine expert from Maharashtra with substantive experience. We used the following indicators for calculating transition probabilities:

1. Total number of high-risk and symptomatic adults who underwent ECG screening at PHC
2. Number of patients referred and underwent a diagnostic test
3. Type of ECG abnormality
4. Type of treatment

The survival rates for each abnormality were derived by applying hazard ratio (11) to the survival rates reported in the published literature for each cardiovascular disorder mentioned in Table 1.

The transition probabilities in the control arm were derived through a systematic search of published literature. Indian data was used for all the transition probabilities except for the survival rate of Action Sequence Conduction Defect, which was obtained globally. In addition to this, due to the unavailability of disorder-specific data on QALY, the cost-effectiveness analysis was done using life years (LYs) saved as an outcome indicator.

To estimate LYs saved, the average age of high-risk adults who underwent the ECG screening was 54.6 years (average age of cohort in the intervention arm) as per the collected data, while that for the control arm was considered 57.5 years as mentioned in the CREATE registry (12). It was assumed that the loss to follow-up of abnormal cases screened was negligible considering that the patients were highly motivated to seek healthcare for their condition in the first place, as they approached the PHCs for treatment. In addition, PHC-MOs were asked to follow-up the cases screened positive for abnormality to ensure that the patients visited higher healthcare centers, underwent diagnostic tests, and were on treatment.

### Sensitivity Analysis and Budget Impact Analysis

One-way Sensitivity analyses were performed to account for uncertainty in model assumptions and to address variability. Sensitivity analysis was performed using low and high absolute estimates for mortality and the cost of treatment. Budget impact analysis (BIA) was performed to estimate the cost for scaling up the ECG program at the District, State, and National levels at 2020 prices. The BIA depicted the budget allocation for the 1st, 2nd, 5th, and 10th year.

## Results

We surveyed 73 patients who screened positive for abnormality to document the type of ECG abnormalities and their further diagnosis.

### Program Cost

The costs incurred toward implementing the program were collected. The cost of the ECG machine has been annualized to estimate the programmatic cost. Details of the program cost are presented in Table 2.

**Table 2:** Details of the program cost in INR (USD).

**Items**	**Units**	**Unit price**	**Annualized cost**
ECG machines	40	70,000.00 (945.95)	4,20,000.00 (5,675.68)
Maintenance and consumables	40	35,000.00 (472.97)	1,40,000.00 (1,891.89)
Expert consultation	12,105	30.00 (0.41)	3,63,150.00 (4,907.43)
Contingency	-	-	75,000.00 (1,013.51)
Training	-	-	75,000.00 (1,013.51)
Shared HR cost	-	-	6,19,777.00 (8,375.36)
**Total**			16,92,927.00 (22,877.39)

The time–motion study was used to estimate shared human resource costs. It was found that an approximate time of 12 min of staff nurses was used toward the ECG program, and its estimated annual cost was 209.38 USD (15,494.43 INR). The annualized cost incurred by the program implementers was estimated to be 1.69 million. With this investment, around 12,105 patients were screened. The calculated cost per case screened amounted to 1.89 USD (139.85 INR). The Table 3 shows various costs that were considered for the purpose of decision-analytic modeling in the intervention and control arm.

**Table 3:** Cost data used to populate the model for high risk population.

**Parameter**	Cost^*^	**Calculation**
**Intervention arm**		
Cost of screening	139.85 (1.89)	Derived from primary data
Out-of-pocket expenditure (OOPE)	63,539 (858.64)	(9)
Cost of treating arrhythmia	1,28,728.85 (1,739.58)	Cost of treatment as per PMJAY package data + OOPE + cost of screening and diagnosis
Cost of treating action sequence defect	3,75,478.85 (5,074.04)	
Cost of treating hypertrophy	1,56,328.85 (2,112.55)	
Cost of treating MI	1,73,478.85 (2344.31)	
Cost of treating other disorders	70,078.85 (947.01)	
**Control arm**		
Cost of treating arrhythmia	1,28,589 (1737.69)	Cost of treatment as per PMJAY package data + OOPE+ cost of diagnosis
Cost of treating action sequence defect	3,75,339 (5072.15)	
Cost of treating hypertrophy	1,56,189 (2110.66)	
Cost of treating MI	1,73,339 (2342.42)	
Cost of treating other disorders	69,939 (945.12)	

* *Cost presented in INR (USD)*.

### Cost-Effectiveness Analysis

Transition probabilities were used to populate the decision-tree model, as shown in Figure 1. Supplementary Table 1 presents transition probabilities.

**Figure 2:**
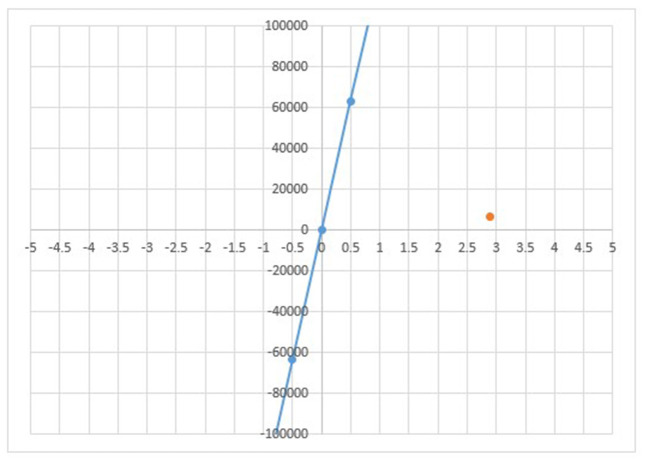
Cost-effectiveness plane in INR. The cost-effectiveness plane depicts ICER (orange dot) lying in the first quadrant because incremental cost of 89.97 USD incurred is saving 2.9 incremental life years. The CER lies below the CE Plane or willingness to pay threshold thus, intervention is cost-effective.

The cost of the intervention arm was 97.07 USD (7,183.64 INR) with 14 life-years saved, whereas the cost incurred in the comparator arm (routine care scenario) was 7.11 USD (526.16 INR) for 11 life-years saved. The ECG screening intervention in primary care has proved to be highly cost-effective for high-risk adult and symptomatic populations, saving around 2.896 life-years at an incremental cost of ~89.97 USD (6,657.47 INR) with ICER of 31.07 USD (2,299.06 INR) per life-year saved (Figure 2).

**Figure 3:**
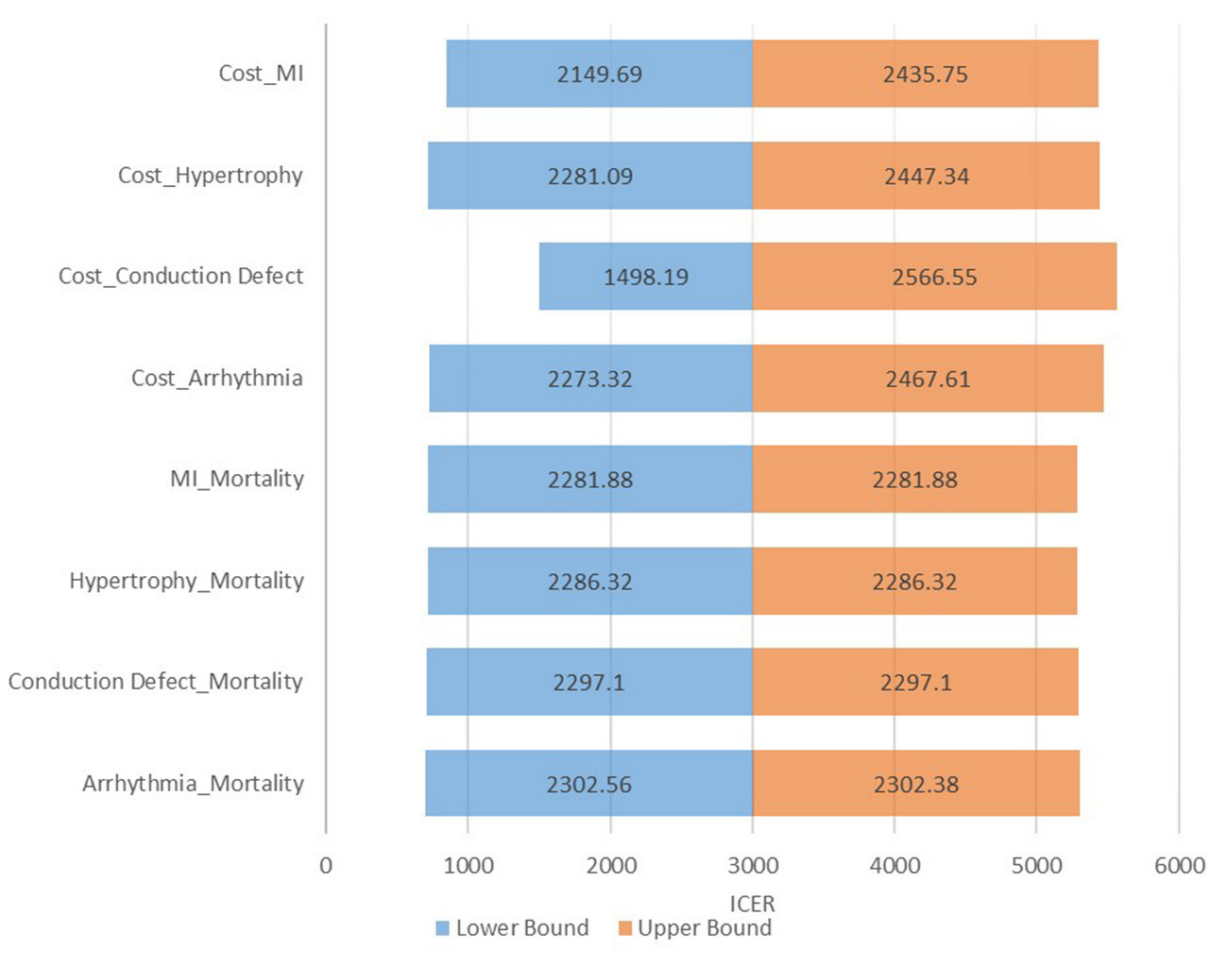
Tornado diagram. The tornado diagram depicts minor variation in ICER after controlling one variable at a time, indicating robustness of the model and its interpretation.

The intervention is cost-effective as the ICER lies well-below the CE Plane or willingness to pay threshold fixed at GDP per capita. The intervention is considered cost-effective if it is <2,009 USD (INR 1,48,666), the GDP per capita of India at 2018 price. The one-way sensitivity analysis indicates that parameters have the most significant effect on ICER when they are varied individually. Figure 3 presents a tornado diagram depicting sensitivity analysis and Table 4 depicts computed sensitivity analysis.

**Table 4:** One-way sensitivity analysis.

**Intervention arm: varying the probabilities of mortality**				
Scenario 1	Using the lower bound mortality for arrythmia		Scenario 2	Using the upper bound mortality for arrythmia
Probability	0.167		Probability	0.25
ICER	2323.56		ICER	2323.56
Scenario 3	Using the lower bound mortality for action sequence conduction defect		Scenario 4	Using the upper bound mortality for action sequence conduction defect
Probability	0.032		Probability	0.048
ICER	2325.37		ICER	2325.37
Scenario 5	Using the lower bound mortality for hypertrophy		Scenario 6	Using the upper bound mortality for hypertrophy
Probability	0.083		Probability	0.125
ICER	2325.67		ICER	2325.67
Scenario 7	Using the lower bound mortality for MI		Scenario 8	Using the upper bound mortality for MI
Probability	0.074		Probability	0.111
ICER	2325.03		ICER	2325.03
**Intervention arm: varying the cost of intervention with lower bounds (assumption: early screening will lead to early diagnosis and reduced cost of care)**				
Scenario 9: Arrhythmia	Cost: 1,15,478.85	ICER	2,294.81	
Scenario 10: Conduction defect	Cost: 70,078	ICER	1,519.68	
Scenario 11: Hypertrophy	Cost: 1,51,728.85	ICER	2,302.58	
Scenario 12: MI	Cost: 1,27,578	ICER	2,171.17	
**Control arm: varying the cost of treatment with upper bounds (assumption: delayed diagnosis may lead to identification in later stage of disease leading**				
to higher cost of care)				
Scenario 9: Arrhythmia	Cost: 2,15,339	ICER	2,320.55	
Scenario 10: Conduction defect	Cost: 4,77,339	ICER	2,320.55	
Scenario 11: Hypertrophy	Cost: 1,94,139	ICER	2,320.55	
Scenario 12: MI	Cost: 2,15,339	ICER	2,293.46	

### Budget Impact Analysis

While performing BIA, the budget of the 1st year incorporated major capital investment required in the 1st year of program scale-up. The budget for the 2nd, 5th, and 10th year depicted the incurred annual implementation cost. In addition, the budget of the 5th year was estimated by considering the need for short refresher training to the health workers.

Supplementary Tables 2a,b provides the budget calculation for 1st, 2nd, 5th, and 10th year of implementation cost in Indian Rupees. The state-wide scale-up cost across 1,474 PHCs in 33 districts of Gujarat for the ECG program is estimated to be around 155.2 million for the 1st year, while the nation-wide scale-up cost was calculated for 24029 PHCs and 720 districts (13) was 2,706 million in the 1st year. This budget was calculated by projecting the annualized cost of implementation in the Ahmedabad district.

## Discussion

Opportunities to screen coronary heart disease and its risk factors are missed at the primary healthcare level (14–16). Project Lifeline, the ECG at PHC level at Ahmedabad, primarily addresses this concern and screens all the high-risk cases for cardiac abnormalities in the primary care setting.

The effectiveness of ECG technology for screening in primary care settings in developed countries is promising (4, 12, 17, 18); however, evidence in the low and middle-income country context is limited. The present work validates the evidence on the cost-effectiveness of ECG screening in a primary care setting in the Indian context when individuals at high risk of developing CVD undergo screening. The cost-effectiveness analysis shows that the ICER lies in the first quadrant of the cost-effective plane, which suggests that an additional cost of 31.07 USD (2,299.06 INR) is incurred for saving one additional life-year making the intervention cost-effective.

Previous cost-effectiveness study using a 12-lead ECG device with the general population concluded the effectiveness of the ECG with an at-risk population (19). Other two studies conducted in young athletes were also cost-effective for pre-participation screening for the study group (18, 20). The findings and recommendations of the present study are consistent with many studies conducted in the past (3–6, 18, 20–23) that recommend ECG screening for high-risk cases (young athletes and elderly and symptomatic adults).

We assumed that with early screening and identification of cardiac abnormality, there might be an initial spurt in the case-load at referral health care centers for seeking care, but it may eventually reduce the burden due to timely management of cases. Thus, active screening of high-risk populations with ECG can be a clinical and cost-effective strategy when introduced at the peripheral level of healthcare. In a population being characterized as high-risk, active screening through ECG can be an effective strategy (5, 6, 17–27). A standardized risk-stratification tool (such as Framingham risk score or SCORE tool) can potentially assist in identifying high-risk populations, and only those identified for high risk should be subjected to ECG screening. The Framingham risk score (28) is an algorithm used to estimate the 10-year cardiovascular risk of an individual. This tool was first developed based on data obtained from the Framingham Heart Study (29). The Systematic Coronary Risk Evaluation (SCORE) is high and low cardiovascular risk charts based on gender, age, total cholesterol, systolic blood pressure and smoking status, with relative risk chart, qualifiers and instructions (30–32).

### Strengths and Limitations of the Study

The study has calculated the cost of screening cardiac abnormalities through ECG devices in the primary healthcare setting in the Indian context. To the best of our knowledge, such a study cumulatively assessing the clinical and cost-effectiveness of the portable ECG device in the primary healthcare setting has not been studied.

For assessing the cost-effectiveness, there were several data gaps in terms of disorder-specific data on QALY, OOPE, and data on the line of treatment in the Indian context. Thus, the cost-effectiveness analysis was performed using LYs saved as an outcome measure. Considering the project is not matured enough, we could not do the follow-up of patients after treatment. Thus, long-term consequences could not be studied. The OOPE for CVD, in general, was considered for modeling. In addition to this, data gap in disorder-specific management such as line of treatment for arrhythmia, action sequence conduction defect, increase in wall thickness of atria and ventricle, myocardial ischemia, and other disorders was sought by consulting a group of experts. More research is recommended for addressing these limitations in the future by taking a larger sample size and longer study duration.

## Conclusion

The use of a 12-lead portable ECG facility to screen cardiac abnormalities among high-risk and symptomatic adults, supported with expert consultation for interpretation of ECG results, is both reasonable in cost and effective at saving lives. The screening facility at the primary health care level may lead to early identification of the disease and prompt management. Further, cost data should be validated on a larger cohort prospectively.

## Data Availability Statement

The original contributions presented in the study are included in the article/Supplementary Material, further inquiries can be directed to the corresponding author/s.

## Ethics Statement

The studies involving human participants were reviewed and approved by Institutional Ethics Committee, Indian Institute of Public Health Gandhinagar. The patients/participants provided their written informed consent to participate in this study.

## Author Contributions

KS, SS, and PK conceptualized the study. CD facilitated data for the study. AP substantially contributed to the acquisition, data analysis, and drafted manuscript. SG, DS, and TP provided technical inputs and critically reviewed the manuscript. All authors contributed significantly to the manuscript and provided approval for publication of the content.

## Funding

This study was part of the Regional Resource Center for Health Technology Assessment, supported by the Department of Health Research, Government of India. The grant no. is F.No.T.11011/08/2017-HR/3136744. The funder was not involved in the study design, collection, analysis, interpretation of data, the writing of this article, or the decision to submit it for publication.

## Conflict of Interest

The authors declare that the research was conducted in the absence of any commercial or financial relationships that could be construed as a potential conflict of interest.

## Publisher's Note

All claims expressed in this article are solely those of the authors and do not necessarily represent those of their affiliated organizations, or those of the publisher, the editors and the reviewers. Any product that may be evaluated in this article, or claim that may be made by its manufacturer, is not guaranteed or endorsed by the publisher.
