# Supplementary material for: Cost-Effectiveness of Portable Electrocardiogram for Screening Cardiovascular Diseases at a Primary Health Center in Ahmedabad District, India
Source: Front Public Health. 2021 Dec 3;9:753443. doi: 10.3389/fpubh.2021.753443 (PMC8678108; doi:10.3389/fpubh.2021.753443)
Supplement: Supplementary file 1 [file Table_1.docx]

**Supplementary Table 1 Transition probabilities used to populate the model for high risk population**

| **Transition from** | **Transition To** | **Transition Probabilities** | **Reference** |
| --- | --- | --- | --- |
| **Intervention Arm** | | | |
| ECG Screening | Screened Positive | 0.04 | Primary Data |
| ECG Screening | Screened Negative | 0.96 | Primary Data |
| Screened Positive | Diagnosed Positive | 0.91 | Primary Data |
| Screened Positive | Diagnosed Negative | 0.09 | Primary Data |
| Diagnosed Positive | Arrhythmia | 0.158 | Primary Data of ECG followed by Expert Opinion |
| Diagnosed Positive | Action Sequence Disorder | 0.211 | Primary Data of ECG followed by Expert Opinion |
| Diagnosed Positive | Hypertrophy | 0.316 | Primary Data of ECG followed by Expert Opinion |
| Diagnosed Positive | Myocardial Infarction and Ischemia | 0.263 | Primary Data of ECG followed by Expert Opinion |
| Diagnosed Positive | Others | 0.053 | Primary Data of ECG followed by Expert Opinion |
| Arrhythmia | Morbidity | 0.776 | Derived on applying Hazard Ratio on probabilities in the control arm |
| Arrhythmia | Mortality | 0.208 |  |
| Action Sequence Disorder | Morbidity | 0.955 |  |
| Action Sequence Disorder | Mortality | 0.040 |  |
| Hypertrophy | Morbidity | 0.886 |  |
| Hypertrophy | Mortality | 0.104 |  |
| Myocardial Infarction and Ischemia | Morbidity | 0.898 |  |
| Myocardial Infarction and Ischemia | Mortality | 0.092 |  |
| Other Disorders | Morbidity | 0.999 |  |
| Other Disorders | Mortality | 0.001 |  |
| **Control Arm** | | | |
| No ECG Screening | Diagnosed Positive | 0.1 | Chauhan & Aeri,2013[13] |
| No ECG Screening | Diagnosed Negative | 0.9 |  |
| Diagnosed Positive | Arrhythmia | 0.3566 | Bodhke et al., 2019[14] |
| Diagnosed Positive | Action Sequence Disorder | 0.07 |  |
| Diagnosed Positive | Hypertrophy | 0.3466 |  |
| Diagnosed Positive | Myocardial Infarction and Ischemia | 0.1966 |  |
| Diagnosed Positive | Others | 0.0302 |  |
| Arrhythmia | Morbidity | 0.786 | Sudan et al., 2018 [15] (Derived Pmorbidity= 1-Pmortality) |
| Arrhythmia | Mortality | 0.214 | Sudan et al., 2018[15] |
| Action Sequence Disorder | Morbidity | 0.959 | Hayashi et al., 2016 [16] (Derived Pmorbidity= 1-Pmortality) |
| Action Sequence Disorder | Mortality | 0.041 | Hayashi et al., 2016 [16] |
| Hypertrophy | Morbidity | 0.893 | Bahl A, 2013[17] (Derived Pmorbidity= 1-Pmortality) |
| Hypertrophy | Mortality | 0.107 | Bahl A, 2013[17] |
| Myocardial Infarction and Ischemia | Morbidity | 0.905 | Sharma & Bhatt, 2018[18] (Derived Pmorbidity= 1-Pmortality) |
| Myocardial Infarction and Ischemia | Mortality | 0.095 | Sharma & Bhatt, 2018 [18] |
| Other Disorders | Morbidity | 0.999 | (Derived Pmorbidity= 1-Pmortality) |
| Other Disorders | Mortality | 0.001 | Derived (Pother = 1- (Parrhythmia+ Pconduction defect+ Phypertrophy + Pmyocardial ischemia) |
| Hazard Ratio | | 0.97 | Lindekleiv et al., 2013[12] |
