# Supplementary material for: Cost-Effectiveness of Portable Electrocardiogram for Screening Cardiovascular Diseases at a Primary Health Center in Ahmedabad District, India
Source: Front Public Health. 2021 Dec 3;9:753443. doi: 10.3389/fpubh.2021.753443 (PMC8678108; doi:10.3389/fpubh.2021.753443)
Supplement: Supplementary file 2 [file Table_2.docx]

**Supplementary Table 2a: Cost Projection for State Level Scale-Up [INR (USD)]**

| **Sr. No.** | **Budget Head** | **Items** | | **Unit Definition** | **Units** | **Unit price** | **Cost at 1^st^ year** | | **Cost at 2^nd^ Year** | | **Cost at 5^th^ Year** | | **Cost at 10^th^ Year** | |
| --- | --- | --- | --- | --- | --- | --- | --- | --- | --- | --- | --- | --- | --- | --- |
| **State Level** | | | | | | | | | | | | | | |
| A | Capital Cost | | ECG Machines | PHC | 1,474 | 79,000  (1068.57) | 11,64,46,000 (157,3594.59) | | - | | - | | - | |
|  |  |  | Training | District | 33 | 84,647  (1143.88) | 27,93,351  (377,47.99) | | - | | 15,20,719  (205,50.26) | | - | |
| **Total (A)** | | | | | | | | **11,92,39,351**  **(161,1342.58)** | | **-** | | **15,20,719**  **(205,50.26)** | | **-** |
| B | Recurrent Cost | | Maintenance and Consumables | PHC | 1,474 | 3,950  (53.38) | 58,22,300  (786,79.73) | | 59,47,479  (803,71.34) | | 63,39,399 (85,667.55) | | 72,02,417 (97,329.96) | |
|  |  |  | Expert Consultation | Individuals | 706 | 34  (0.46) | 24,004  (324.38) | | 24,520  (331.35) | | 26,136 (353.19) | | 29,694  (401.27) | |
|  |  |  | Contingency | District | 33 | 84,647  (1143.88) | 27,93,351  (377,47.99) | | 28,53,408  (385,59.57) | | 30,41,438  (41,100.51) | | 34,55,487  (466,95.77) | |
|  |  |  | Human Resource Cost | District | 33 | 6,99,495  (9452.64) | 2,30,83,335  (311,936.96) | | 2,35,79,627 (318,643.61) | | 2,51,33,446 (339,641.16) | | 2,85,55,007 (385,878.47) | |
|  |  |  | Additional Cost at Tertiary Care (Including Diagnosis and Management | Individuals | 642 | 6,720  (90.81) | 43,15,104  (583,12.22) | | 44,07,879 (59,565.93) | | 46,98,343 (63,491.12) | | 53,37,956  (721,34.54) | |
| **Total (B)** | | | | | | | | **3,60,38,094**  **(487,001.27)** | | **3,68,12,913**  **(497,471.80)** | | **3,92,38,762 (530,253.54)** | | **4,45,80,561**  **(602,440.01)** |
| **Grand Total** | | | | | | | | **15,52,77,445**  **(209,8343.85)** | | **3,68,12,913**  **(497,471.80)** | | **4,07,59,481 (550,803.80)** | | **4,45,80,561**  **(602,440.01)** |

**Supplementary Table 2b: Cost Projection for National Level Scale-Up [INR (USD)]**

| **Sr. No.** | **Budget Head** | **Items** | | **Unit Definition** | **Units** | **Unit price** | **Cost at 1^st^ year** | **Cost at 2^nd^ Year** | **Cost at 5^th^ Year** | **Cost at 10^th^ Year** |  |
| --- | --- | --- | --- | --- | --- | --- | --- | --- | --- | --- | --- |
| **National Level (2020 Prices)** | | | | | | | | | | | |
| A | Capital Cost | | ECG Machines | PHC | 24,049 | 79,000  (1068.57) | 1,89,98,71,000 (256,739,32.43) | - | - | - |  |
|  |  |  | Training | District | 720 | 84,647  (1143.88) | 6,09,45,840 (823,592.43) | - | 3,31,79,326 (448,369.27) | - |  |
| **Total (A)** | | | | | | | **1,96,08,16,840** (264,97524.86) | **-** | **3,31,79,326** (448,369.27) | **-** | |
| B | Recurrent Cost | | Maintenance and Consumables | PHC | 24,049 | 3,950  (53.38) | 9,49,93,550 (128,3696.62) | 9,70,35,911 (131,128.53) | 10,34,30,257 (1,397,706.18) | 11,75,10,812 (1,587,983.95) |  |
|  |  |  | Expert Consultation | Individuals | 14,017 | 34  (0.46) | 4,76,578 (6440.24) | 4,86,824 (6,578.70) | 5,18,905 (7,012.23) | 5,89,546 (7,966.84) |  |
|  |  |  | Contingency | District | 720 | 84,647  (1143.88) | 6,09,45,840  (823,592.43) | 6,22,56,176 (841,299.68) | 6,63,58,651 (896,738.53) | 7,53,92,436 (1,018,816.70) |  |
|  |  |  | Human Resource Cost | District | 720 | 6,99,495  (9452.64) | 50,36,36,400  (680,5897.30) | 51,44,64,583 (6,952,224.09) | 54,83,66,095 (7,410,352.64) | 62,30,18,325 (8,419,166.55) |  |
|  |  |  | Additional Cost at Tertiary Care (Including Diagnosis and Management) | Individuals | 12,755 | 6,720  (90.81) | 8,57,16,307  (115,8328.47) | 8,75,59,208 (1,183,232.54) | 9,33,29,069 (1,261,203.64) | 10,60,34,492 (1,432,898.54) |  |
| **Total (B)** | | | | | | | **74,57,68,675**  **(100,779,55.07)** | **76,18,02,702 (10,294,631.11)** | **81,20,02,977 (10,973,013.20)** | **92,25,45,611 (12,466,832.58)** | |
| **Grand Total** | | | | | | | **2,70,65,85,515 (365,754,79.93)** | **76,18,02,702 (10,294,631.11)** | **84,51,82,302 (11,421,382.46)** | **92,25,45,611 (12,466,832.58)** | |
